# Primary Adenoma Arising From Two Ipsilateral Supernumerary Inferior Parathyroid Adenomas

**DOI:** 10.1210/jcemcr/luad113

**Published:** 2023-09-20

**Authors:** Marine Bolliet, Binit Katuwal, Ramachandra Kolachalam

**Affiliations:** Department of Surgery, Ascension Providence Hospital-Michigan State University College of Human Medicine, Southfield Campus, Southfield, MI 48075, USA; Department of Surgery, Ascension Providence Hospital-Michigan State University College of Human Medicine, Southfield Campus, Southfield, MI 48075, USA; Department of Surgery, Ascension Providence Hospital-Michigan State University College of Human Medicine, Southfield Campus, Southfield, MI 48075, USA

**Keywords:** primary hyperparathyroidism, parathyroid adenoma, parathyroidectomy, supernumerary parathyroid adenoma

## Abstract

A single parathyroid adenoma is the most common cause of primary hyperparathyroidism (PHPT). However, multiple synchronous adenomas can be found at surgery. More uncommon are ipsilateral synchronous adenomas, and that combined with a supernumerary gland, is even more rare. Here we present a case of PHPT due to an ipsilateral double adenoma of the inferior parathyroid gland, which was supernumerary. The diagnosis was made preoperatively by ultrasonography; however, sestamibi scan showed only a single hyperfunctioning gland on the left side. This was further substantiated by the use of intraoperative parathyroid hormone (PTH) monitoring, wherein PTH levels decreased to less than 50% of preoperative values only after the complete removal of the second adenomatous gland. This case report highlights the importance of preoperative localization and intraoperative PTH monitoring in evaluating patients with PHPT in the setting of multiple synchronous parathyroid adenoma.

## Introduction

Primary hyperparathyroidism (PHPT) is a common condition affecting 1 to 7 per 1000 patients, with a prevalence of around 0.83% in a United States–-based study. The incidence of the condition is up to 100 000 patients per year in the United States [1]. Parathyroid pathologies are also well described, including adenomas, hyperplasia, and malignancies, with adenomas being by far the most common. Parathyroid glands are typically described as being 4 in total, 2 superior, and 2 inferior, on either side. It is well described that parathyroid glands often have aberrant locations, commonly in the thymic tissue, tracheoesophageal groove, and even the carotid sheath, among others. The exact location of where the aberrant gland is located depends on whether the gland is superior or inferior. A significant proportion of patients have more than 4 parathyroid glands, also called supernumerary glands, with incidences reported to be from 13% to 15% [2]. However, only a single gland is found as the culprit when an adenoma is identified. Synchronous adenomas, although initially considered to represent asymmetric hyperplasia, have been known to exist. Several studies have since confirmed the presence of multiple distinct adenomas as a separate diagnosis from parathyroid hyperplasia. A literature review in 2015 demonstrated that when multiple parathyroid adenomas were identified, they were often contralateral to the sestamibi scan–localized adenoma [3]. There are very few reported cases of multiple parathyroid glands on the same side that are both found to be primary parathyroid adenomas. Even more rare is the presence of an ipsilateral double adenoma including an adenoma of a supernumerary gland [4].

We present an asymptomatic 70-year-old woman with PHPT with decreased bone density requiring surgical intervention. The preoperative evaluation was suspicious for parathyroid adenoma of 2 adjacent enlarged glands at the left inferior location.

## Case Presentation

The patient is a 70-year-old woman without any previous history of cancers who presented to the office with hypercalcemia detected during routine biochemical testing.

## Diagnostic Assessment

**Figure 1.**
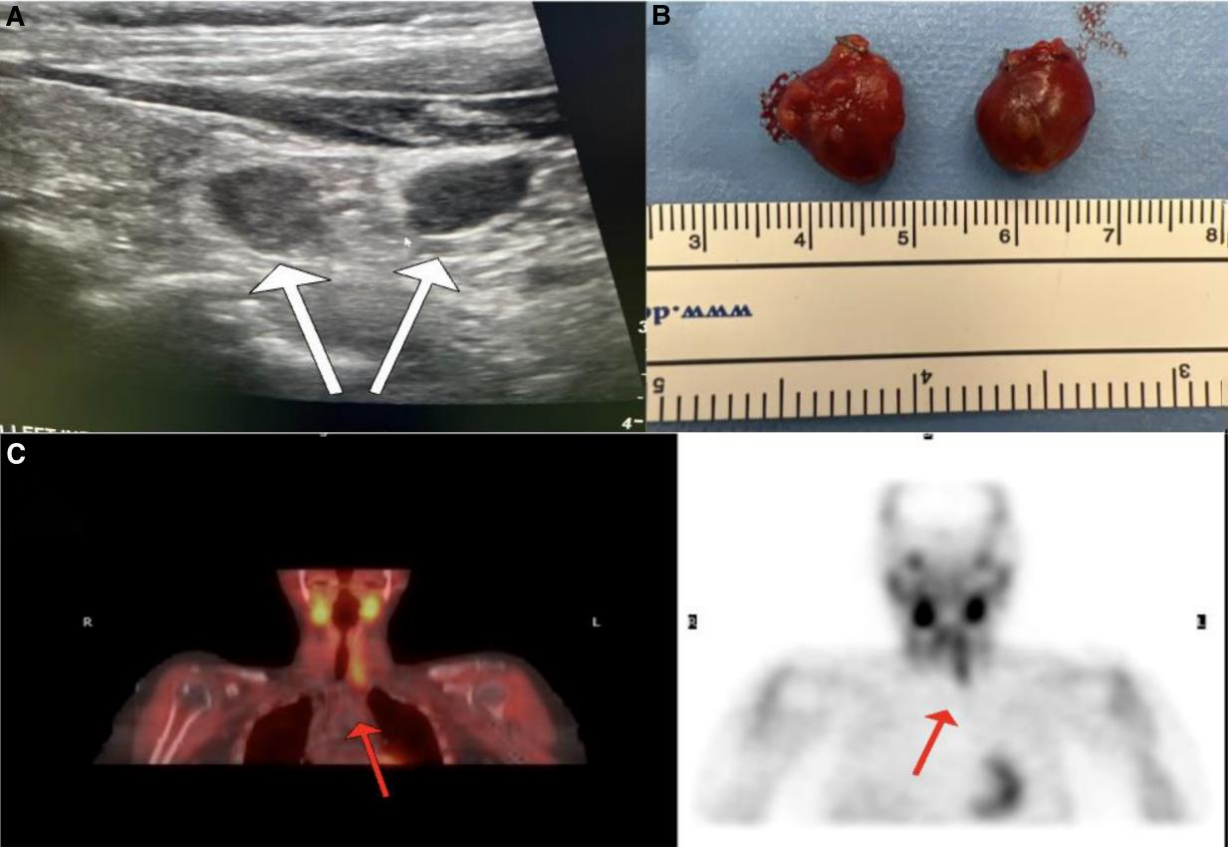
A, Ultrasound of the neck demonstrating 2 inferior parathyroid adenomas. B, The resected inferior parathyroid glands (gland 1: 1.6 × 1.1 × 0.6 cm and 0.5 g; gland 2: 1.5 × 1.1 × 0.4 cm and 0.57 g. C, Preoperative sestamibi scan demonstrating only one hyperfunctioning gland, left inferior parathyroid.

She underwent a standard workup including measurement of parathyroid hormone (PTH) levels, which were found to be elevated at 72.1 pg/mL (15-65 pg/mL; 7.64 pmol/L). Bone density scan had demonstrated high fracture risk with a T-score of −3.0. A preoperative parathyroid ultrasound of the neck showed 2 adjacent hypoechoic nodules in the left lower neck (Fig. 1), highly suggestive of parathyroid adenomas. However, sestamibi scan showed only one hyperfunctioning gland corresponding to the left inferior parathyroid gland (see Fig. 1).

## Treatment

**Figure 2.**
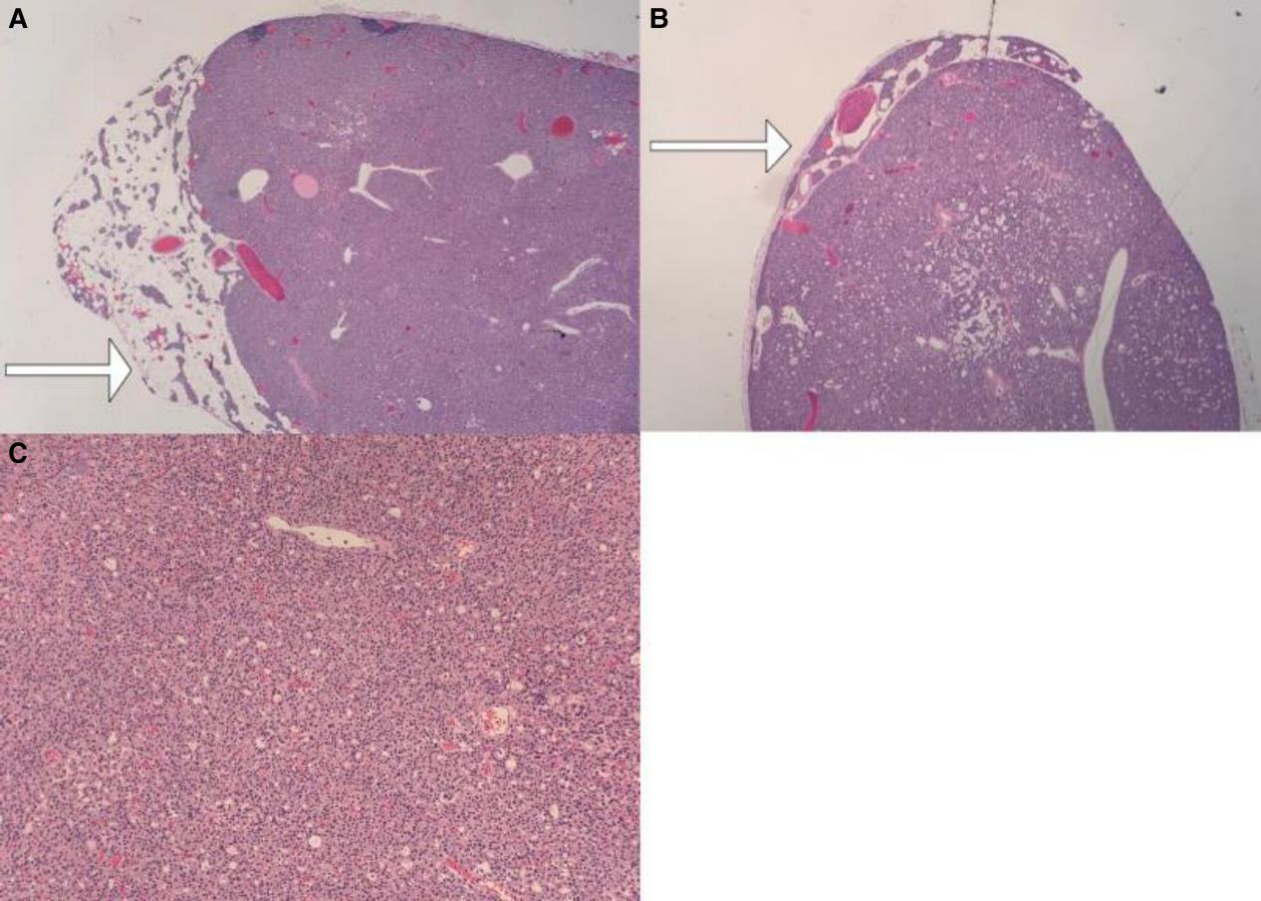
Pathological examination of both inferior parathyroid glands demonstrating a rim of normal parathyroid outside A and B, adenoma capsule with C, compact cells, consistent with adenoma.

Surgery was scheduled for an anticipated left inferior parathyroidectomy. The preoperative PTH level was 160.1 pg/mL (16.97 pmol/L). A second PTH level, 10 minutes after the removal of gland 1, resulted in 115.0 pg/mL (12.19 pmol/L). Due to the failure of intraoperative PTH level to drop less than 50%, further neck exploration was performed that yielded a second enlarged left inferior parathyroid gland (gland 2) (see Fig. 1), consistent with the preoperative ultrasound findings. PTH levels 5 and 10 minutes after removal of gland 2 were 61 pg/mL (6.46 pmol/L) and 48 pg/mL (5.08 pmol/L), respectively. Since the PTH level dropped 50% from the preoperative PTH level, the surgery was concluded. The left superior parathyroid gland was identified and preserved. The patient recovered postoperatively without complications. Histopathology confirmed the presence of adenoma in both glands (Fig. 2).

## Outcome and Follow-up

The patient had laboratory samples drawn 5 months postoperatively that demonstrated a PTH level of 46.8 pg/mol (4.96 pmol/L) and a calcium level of 10.3 mg/dL (8.4-10.5 mg/dL; 2.57 mmol/L). She is doing well and has recovered from the surgery without issues.

## Discussion

PHPT is a common endocrine disorder characterized by excessive secretion of PTH, causing hypercalcemia. The majority of the cases are due to a parathyroid adenoma (80%-85%), with a small fraction of parathyroid hyperplasia (10%-15%), and rarely, parathyroid carcinomas (<1%).

Evaluation of patients with PHPT includes measurement of serum and urine calcium and PTH levels. If the diagnosis of PHPT is confirmed, localization of the parathyroid adenoma is essential in planning for surgery. Preoperative evaluation should be conducted with scintigraphy imaging (usually a sestamibi scan) that should be followed up by either ultrasonogram or 4-dimensional–computed tomography/magnetic resonance imaging [3]. However, the sensitivity and specificity of preoperative imaging, especially the sestamibi scan, drops substantially in the presence of multigland involvement to a degree that preoperative assessment alone becomes unreliable [3]. Another preoperative consideration is genetic testing. The role of genetic testing has been studied and indications for genetic testing for syndromes, such as multiple endocrine neoplasia, have been described [3]. Major criteria include hyperparathyroidism, pituitary adenoma, and pancreatic tumors. The presence of 2 major criteria is an indication for genetic testing. With this patient, while she had hyperparathyroidism only, she had 2 separate adenomas. The role of genetic testing is not well defined in these circumstances, but given she had 2 separate adenomas leading to her hyperparathyroidism, it could be argued that she meets the major criteria. Genetic testing was offered to her. Given she had no other symptoms consistent with multiple endocrine neoplasia syndromes and no known family history, she declined genetic testing at that time.

Intraoperative evaluation using intraoperative PTH monitoring becomes an essential part of the evaluation for multigland disease. Other modalities that have been used for the intraoperative identification of parathyroids are indocyanine green and near-infrared autofluorescence [5]. There are some instances for which preoperative and intraoperative internal jugular venous sampling has been performed to help lateralize parathyroid adenomas [3]. The utility of venous sampling is emphasized in reoperations where significant scarring can lead to inconclusive preoperative imaging.

Multiple models have been tested to evaluate for the presence of multiglandular disease both preoperatively and intraoperatively. Two specific predictability models worth mentioning are the Wisconsin Index (WIN) and the Yale Prediction Model [6, 7]. The WIN was defined as the multiplication of preoperative serum calcium by preoperative PTH. Patients were divided into 3 WIN categories: low (<800), medium (801-1600), and high (>1600), with probability of multigland involvement in 61% if the WIN score was greater than 1600 [6]. Similarly, the Yale Model is an intraoperative model based on preoperative, intraoperative (preexploration, time 0, every 5 minutes post resection), and postoperative PTH and calcium levels, with the accuracy of the model close to 95% [7].

Adenomas are usually solitary; however, they can also disguise as double adenomas. Double adenomas are known to be present in up to 15% of all PHPT patients [8]; however, finding an adenoma in a supernumerary gland is rare. This patient had 1 superior and 2 inferior glands on the left side.

Studies have been conducted previously to identify the relative incidences of double adenomas. A study by Milas et al [8] found that double adenoma has a predilection to be superior and bilateral in about 45% of cases. Similarly, a study by Abboud et al [9] showed that double adenoma can be present in multiple configurations—both superior, both inferior, both right, both left, right superior and left inferior, and left superior and right inferior—with a preference of crossed bilateral distribution.

Double adenoma represents a truly separate entity that can contribute to confusion and pitfalls in the management of PHPT. There have been studies trying to differentiate adenoma from parathyroid hyperplasia; however, controversies still exist. Clinicopathological differentiation has been attempted between these 2 entities. The presence of a rim of normal parathyroid tissue adjacent to an encapsulated nodule has been the “gold standard” for the diagnosis of a parathyroid adenoma along with the other features such as a fibrous capsule, cellular pleomorphism, presence of nodules, and mitotic figures [10]. Adenomas are monoclonal lesions arising from a single mutated precursor cell, while multiglandular disease, or hyperplasia, is likely polyclonal, suggesting that they represent 2 different disease processes [10].

Clinically, patients with PHPT due to adenoma respond well to localized gland excision, compared to patients with hyperplasia, who require 4-gland exploration. It is critical to be aware of the relationships between the clinical diagnosis and the appropriate response to surgical intervention. In the context of double adenomas, where resection of the first enlarged gland does not drop intraoperative PTH levels, clinical suspicion for an additional adenoma should be high prior to a complete 4-gland exploration. This highlights the importance of intraoperative PTH monitoring, especially in cases where double adenomas are present.

## Learning Points

- Parathyroid adenoma is a common cause of PHPT.
- Anatomic and pathologic variations do exist and should be kept in mind during the evaluation of the condition.
- Preoperative and intraoperative localization techniques should be liberally used for accurate identification of the problem.
- Intraoperative PTH level monitoring and a drop of more than 50% from preoperative level in 10 minutes is considered indicative of adenoma removal.

## Data Availability

Data sharing is not applicable to this article as no data sets were generated or analyzed during the current study.

## Abbreviations

PHPT: primary hyperparathyroidism
PTH: parathyroid hormone
WIN: Wisconsin Index
